# Finding a Needle in the Virus Metagenome Haystack - Micro-Metagenome Analysis Captures a Snapshot of the Diversity of a Bacteriophage Armoire

**DOI:** 10.1371/journal.pone.0034238

**Published:** 2012-04-11

**Authors:** Jessica Ray, Michael Dondrup, Sejal Modha, Ida Helene Steen, Ruth-Anne Sandaa, Martha Clokie

**Affiliations:** 1 Department of Biology, University of Bergen, Bergen, Norway; 2 Uni Research AS, Uni Computing CBU, Bergen, Norway; 3 Department of Infection, Immunity and Inflammation, University of Leicester, Leicester, United Kingdom; Universidad Miguel Hernandez, Spain

## Abstract

Viruses are ubiquitous in the oceans and critical components of marine microbial communities, regulating nutrient transfer to higher trophic levels or to the dissolved organic pool through lysis of host cells. Hydrothermal vent systems are oases of biological activity in the deep oceans, for which knowledge of biodiversity and its impact on global ocean biogeochemical cycling is still in its infancy. In order to gain biological insight into viral communities present in hydrothermal vent systems, we developed a method based on deep-sequencing of pulsed field gel electrophoretic bands representing key viral fractions present in seawater within and surrounding a hydrothermal plume derived from Loki's Castle vent field at the Arctic Mid-Ocean Ridge. The reduction in virus community complexity afforded by this novel approach enabled the near-complete reconstruction of a lambda-like phage genome from the virus fraction of the plume. Phylogenetic examination of distinct gene regions in this lambdoid phage genome unveiled diversity at loci encoding superinfection exclusion- and integrase-like proteins. This suggests the importance of fine-tuning lyosgenic conversion as a viral survival strategy, and provides insights into the nature of host-virus and virus-virus interactions, within hydrothermal plumes. By reducing the complexity of the viral community through targeted sequencing of prominent dsDNA viral fractions, this method has selectively mimicked virus dominance approaching that hitherto achieved only through culturing, thus enabling bioinformatic analysis to locate a lambdoid viral “needle" within the greater viral community “haystack". Such targeted analyses have great potential for accelerating the extraction of biological knowledge from diverse and poorly understood environmental viral communities.

“Before beginning a Hunt, it is wise to ask someone what you are looking for before you begin looking for it." - Winnie the Pooh

## Introduction

### Blurb

Targeted metagenome analysis identifies lambdoid virus and reveals sub-genomic viral diversity in an Arctic hydrothermal plume.

Viruses are the most numerous and diverse biological entities in the oceans [1], [2], [3], [4] with important roles in biochemical cycling and in structuring microbial communities [5], [6], [7]. Not only do viral infection and lysis influence the structure of prokaryote communities, they also control the partitioning of nutrient fluxes “up" the food chain via predation and “down" the food chain to the pool of dissolved organic material (DOM) [8], [9], [10], [11], [12], [13], [14]. Viruses also have the ability to manipulate the life history and evolution of the host community by acting as major conduits of genetic exchange, tranducing an estimated 10^16^–10^19^ Gbp of DNA per year in the global ocean [4], [15]. Several examples exist where viral infection has introduced beneficial traits to the host that can be acted upon by natural selection [7], [16].

Deep-sea hydrothermal vent environments are one of the most physically and chemically diverse biomes on Earth, characterised by chemically enriched seawater vented from fissures in volcanic sea floor edifices at temperatures up to 400°C [17], [18]. The geothermal reducing power of vent fluid is dispersed from the vent source in plumes that may travel hundreds of kilometres from their source [19]. In these plumes, biological productivity is primarily driven by chemolithoautotrophs, but a considerable portion of the microbial biomass is likely to consist of heterotrophic prokaryotes [17], [20], [21], [22], [23]. Based on our knowledge of other prokaryote dominated communities, the abundance and diversity of prokaryotes in hydrothermal plumes is likely to co-exist with an abundant and diverse virus community. Although significant efforts have been made to describe the diversity and function of plume-derived prokaryotic communities [24], [25], [26], [27], little focus has been given to the viral component in these systems.

Viral abundance and diversity are intricately linked to the structure of host microbial communities [14], and characterising the host-virus interactions is essential for our understanding of the ecology and functioning of the microbial community. Laboratory studies have shown that fitness-driven adaptation can partially explain the genetic diversification of individual hosts and their cognate viruses [28], [29], [30]. However, unravelling the enormous complexity of host-virus pairs and their ecological significance in heterogeneous marine environments has proven to be a formidable challenge. The dominant host archea/bacteria may either not be known, or, if they have been identified using PCR based or metagenomic approaches, they may not be amenable to culture. Indeed, many marine micro-organisms that are abundant in metagenomic datasets are commonly absent from culture collections [31], confounding the isolation of viruses from marine environments. The quandary then remains of how to identify key viruses in marine environments.

Targeted analysis of marine viral communities using second-generation sequencing technologies has generated a wealth of sequence data [32], [33], [34] that can be mined with bioinformatics tools [35], [36] to probe viral diversity in marine samples in the absence of cultured specimens. This culture-independent approach has two disadvantages. Firstly, the sheer diversity of viruses in water samples complicates read assembly and annotation and results in very few near complete genomes being assembled [32], [37], [38]. Secondly, there is an under-representation of reference viral genomes of marine origin in the sequence databases [33], which impedes conclusive identification of marine virus sequences that are either divergent from known viral genes or are completely novel [37], [38].

We present a novel method which utilizes the inherent advantages of both culture-dependent and culture-independent studies in order to examine the composition of dominant viral populations collected from hydrothermal plume and surrounding seawater proximal to the Loki's Castle vent field on the Arctic Mid-Ocean Ridge [39]. The viral genomes present in virus concentrates from each sample were separated by pulsed field gel electrophoresis (PFGE) [40], and DNA from the dominant PFGE bands was extracted, amplified and subjected to metagenomic deep sequencing. In this way, we have exploited the power of high-throughput sequencing to analyse an unknown viral community, while at the same time decreasing the complexity of the viral population under investigation such that the resulting metagenome more closely approximates single-strain dominance obtainable through culturing or in extreme environments [41]. This specificity allowed the culture-independent identification of an almost complete lambda-like phage genome from a key viral fraction of the hydrothermal plume. From our data we have also identified diverse virus-like sequences that may serve as a genetic signature of the host-virus “arms race" in this poorly-understood marine ecosystem. These methods and analyses provide a valuable snapshot of the biology and diversity within populations of viruses present at the time of sampling, and also shed light upon the potential fitness-driven adaptive strategies present in an undescribed deep sea virus community.

## Results

### Prokaryote and viral abundance

Flow cytometric analysis of prokaryote and viral abundance in the Loke hydrothermal plume at the time of sampling for metagenome analysis revealed 1.0×10^5^ prokaryotes ml^−1^ and 3.9×10^5^ VLPs ml^−1^. The surrounding seawater had similar values with 9.4×10^4^ prokaryotes ml^−1^ and 3.4×10^5^ VLPs ml^−1^.

### Analysis of viral community composition

The PFGE analysis revealed that the genome size of the prominent members of the viral community from both sample sites was between 31 and 48 kb (Figure S1). The dominant PFGE bands were excised and sequenced, resulting in 66,208 sequencing reads from the plume sample and 81,017 from the surrounding seawater sample. The sequence reads had an average length of 268±117 bp and 316±139 bp, respectively (Table 1). BLAST analysis was carried out for all unassembled 454 reads against three NCBI databases: the non-redundant protein sequence database (nr), non-redundant environmental samples (env_nr), and a nucleotide boutique database of all viral RefSeq genomes (virus_refseq). The results from the analysis are summarised in Table 1, where it can be seen that ∼44% of reads for the plume sample and ∼55% of reads from the surrounding seawater sample had at least one hit to any of the four databases searched. For both the plume and surrounding seawater metagenomes, the environmental database (env_nr) yielded the greatest number of hits (BLASTX, *E*-value
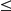
10^−3^) (Altschul et al 1997). The second greatest number of hits to entries was to the nr database, which gave hits for 15235 reads from the plume sample (23% of total reads) and 17168 reads from the surrounding seawater sample (21% of total reads) (Genbank Sequence Read Archive SRP005853). The viral RefSeq database yielded the fewest number of hits for both the plume and surrounding seawater samples, with 5518 and 7493 hits (8.3% and 9.2% of total reads), respectively.

**Table 1:** Sequence data metrics and summary statistics for BLAST database searches.

		Loki's Castle plume		Surrounding seawater	
		number	percentage	number	percentage
**Sequence data**	total reads	66208	100	81017	100
	total bases	17783483		25648368	
	longest read length	607		639	
	shortest read length	40		40	
	median read length	273		336	
	mean read length ± s.d.	268±117		316±139	
	GC content %		41.9		39.1
**Sequences with hits to databases**	non-redundant (NR)	15235	23.01	17168	21.19
	environmental non-redundant (ENR)	26888	40.61	44194	54.55
	RefSeq-Viral (RV)	5518	8.33	7493	9.25
	NR+ENR	13102	19.79	16067	19.83
	NR+RV	4424	6.68	6286	7.76
	ENR+RV	4165	6.29	7102	8.77
	NR+ENR+RV	3598	5.43	6154	7.60
**Sequences with hit to at least one database**	NR+ENR+RV+environmental (ENV)	29548	44.63	45554	56.23
**Sequences with no hits to any database**	NR+ENR+RV+environmental (ENV)	36660	55.37	35463	43.77
**Taxonomic classification with MEGAN (BLASTX E< = 0.001)**	Viruses	1205	1.82	2337	2.88
	Eukarya	867	1.31	1253	1.55
	Bacteria	10358	15.64	9118	11.25
	Archaea	438	0.66	627	0.77
	Total	12868	19.44	13335	16.46

### Taxonomic classification

**Figure 1:**
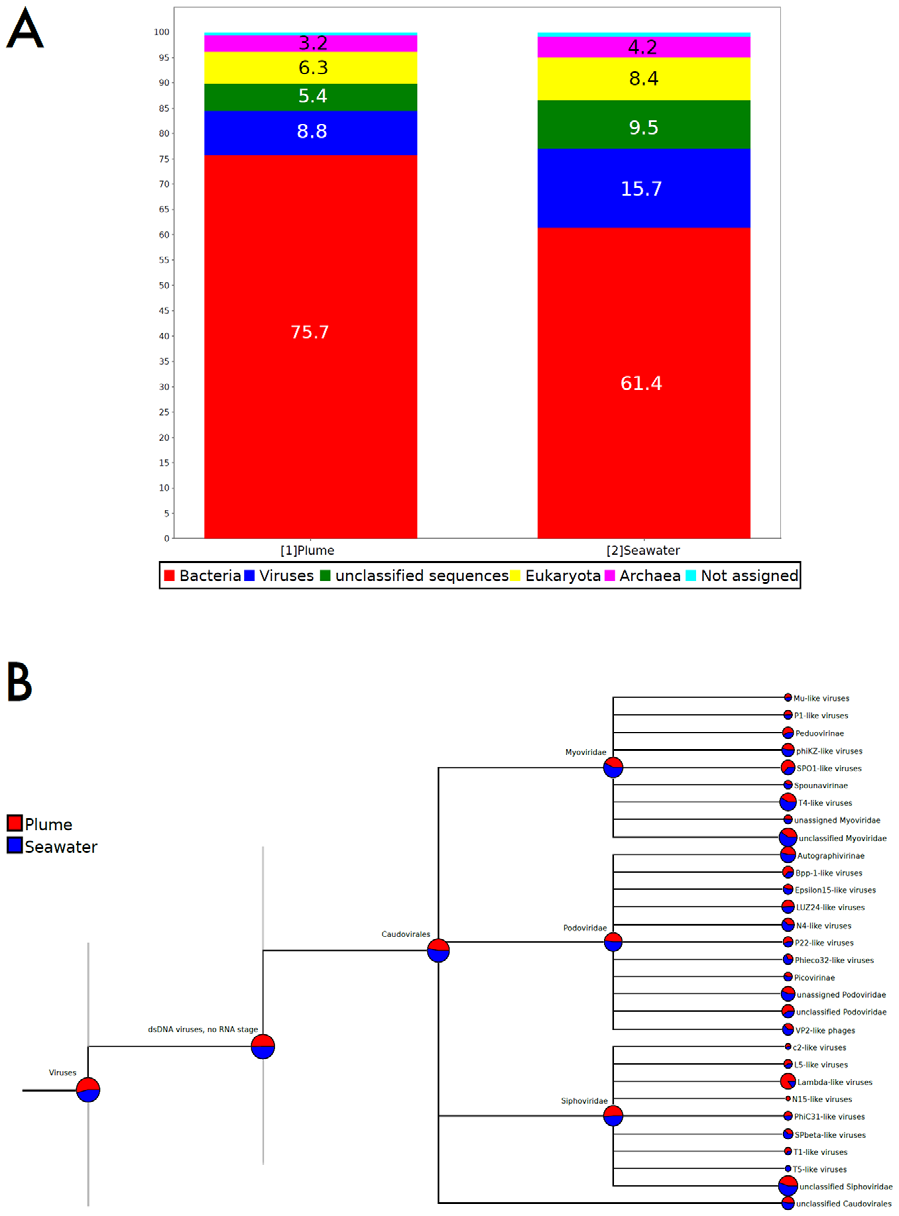
Taxonomic composition of genome sequences as determined by similarity to reference database DNA and protein sequences. (A) Kingdom-level taxonomic assignment of metagenome sequencing reads using a BLASTX search against the NCBI non-redundant (nr) database (E-value≤0.001). (B) Megan4 tree demonstrating relative taxonomic composition of plume (red) surrounding deep-sea (blue) virus communities as determined by TBLASTX similarity (E-value≤0.001) of sequencing reads to known virus genome sequences in the RefSeq Viral Genomes database.

BLASTX analysis of metagenomic sequences against the nr database (E-value
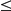
10^−3^) enabled kingdom-level taxonomic assignment of 19.4% (12868 reads) and 16.5% (13335 reads), respectively, of plume and surrounding seawater sequencing reads (Table 1). From these classifiable reads, we identified sequences of viral, bacterial, archaeal and eukaryote origin (Table 1 and Figure 1A). Virus-like sequences accounted for 7.9% and 13.6% of classifiable reads from the plume and surrounding seawater samples, respectively. Bacterial, archaeal and eukaryote sequences accounted for 68.0%, 2.9% and 5.7% of classifiable reads, respectively, in the plume sample, while in the seawater sample they accounted for 53.1%, 3.7% and 7.3% of classifiable reads, respectively.

Virus-like sequences from the plume and surrounding seawater metagenomes were identified by a TBLASTX (E-value< = 10^−3^) search against the virus_refseq database, and the results were analysed using MEtaGenome ANalyzer (MEGAN4). Of the assigned virus-like sequences, however, the majority for both the plume (81.2%) and surrounding seawater (91.5%) samples most closely resembled double-stranded DNA (dsDNA) viruses with no RNA stage (Figure 1B). Of these dsDNA virus-like sequences, 60.2% and 67.1% from the plume and surrounding seawater samples, respectively, were assigned to the order Caudovirales, while the remaining dsDNA virus-like sequences were sparsely distributed among over 20 other viral orders. The diversity of virus-like sequences within the Caudovirales order was dominated approximately equally by Myovirus- (36.4% and 46.3% in the plume and surrounding seawater samples, respectively) and Siphovirus-like (44.2% and 37.4% in the plume and surrounding seawater samples, respectively) sequences, with smaller relative fractions of sequences assigned to Podoviridae (17.4% and 17.0% for plume and surrounding seawater samples, respectively) or to unclassified Caudovirales (2% for both samples).

**Figure 2:**
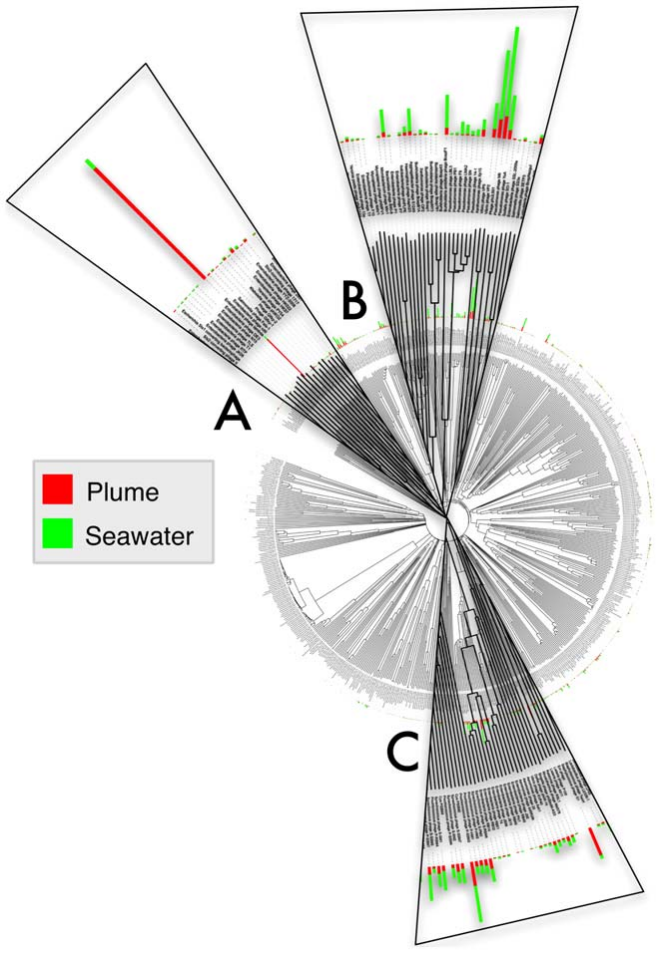
Classification of metagenome sequence reads by best-hit mapping to the Phage Proteomic Tree. Red bars; plume sample. Green bars; surrounding seawater sample. A; Indicates PPT taxon with best blast hits to Enterobacteria phage lambda, B; PPT taxon with hits to other siphoviruses including Pseudomoans phage M6 and Phage phiJL001, and C; PPT taxon with highest hits to different cyanomyoviruses.

Mapping of all bacteriophage-like reads from the plume and surrounding seawater sample metagenomes to the Phage Proteomic Tree [42] using only best BLAST hits allows one to view the taxonomic composition from a slightly different angle compared to MEGAN's least common ancestor approach. This analysis revealed that the samples differ both in the distribution and abundance of hits to different sequenced phage genomes (Figure 2). Phage-like reads were abundant in both metagenomes, although the surrounding seawater virome had a higher frequency of hits to the Phage Proteomic Tree than the plume virome, indicating greater similarity of the viral sub-community in the surrounding seawater sample to sequenced phage genomes (Figure 2). The Phage Proteomic Tree taxon with the greatest number of hits from our datasets was Enterobacteria phage lambda, similarity to which was prominent among plume sample reads, but also present in the surrounding seawater dataset (Figure 2A). An abundance of metagenomic reads also mapped to other siphoviruses such as Pseudomonas phage M6 and Phage phiJL001 (Figure 2B). A third group, consisting of reads with highest similarity to different cyanomyoviruses, exhibited greatest abundance in the surrounding seawater sample (Figure 2C).

Sub-family diversity of virus-like reads varied dependent upon the viral family examined. Within the Myoviridae (Figure 1B), for example, the greatest percentage of reads were unclassified for both the plume (47.1%) and surrounding seawater samples (54.3%), although both samples shared high relative percentages of T4-like viruses (30.9% and 32.7%, respectively, for plume and surrounding seawater samples). Unclassified Podovirales-like reads accounted for only 13.9% and 16.2% of plume and seawater reads, respectively (Figure 1B). Significant similarity to nine other Podoviridae taxa was approximately equally distributed for both the plume and surrounding seawater samples, both datasets of which were dominated by Autographvirinae-like reads (34.0% and 35.5%, respectively) (Figure 1B).

Because the Siphoviridae family of viruses includes many temperate bacteriophage [43], and because previous studies of viral communities in and near hydrothermal vent systems have identified high relative rates of viral lysogeny at these sites [44] and in deep oceans in general [45], we decided to more closely examine the diversity of metagenome reads with highest similarity to the Siphoviridae family of viruses to investigate whether our data contains evidence to support the notion that viral lysogeny is also prevalent in the Loki's Castle hydrothermal plume or surrounding seawater (Figure 1B). Sub-family examination of Siphovirus-like reads demonstrated 70.1% and 91.7% unknown sequences, respectively, from the plume and seawater samples (Figure 1B, bottom row). This examination, together with best-hit mapping to the Phage Proteomic Tree (Figure 2), immediately indicated a difference in relative abundances of Enterobacteria phage lambda-like reads between the plume (Figure 1B) and surrounding seawater samples (Figure 1B). More specifically, lambda-like virus reads accounted for 26.9% of Siphovirus reads in the plume sample, while they only accounted for 5.0% in the surrounding seawater sample. In addition, lambda-like viruses were the only Siphoviruses both with considerable representation in both datasets and with clear differences in relative abundance (>5-fold) between the plume and surrounding seawater samples.

### Mapping the metagenome sequences to the phage lambda genome

**Figure 3:**
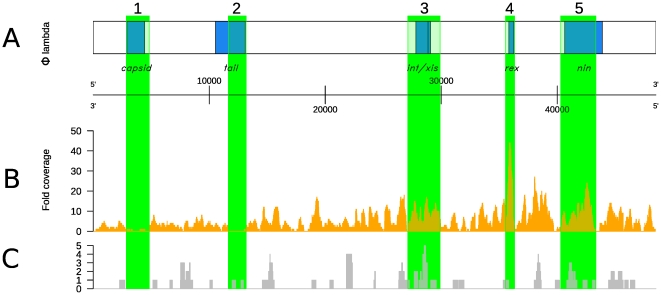
Identification of an Enterobacteria phage lambda-like phage in the two metagenome datasets. (A) Schematic map of the linear lambda genome, modified from [111]. (B) Mapping of plume metagenome reads to the lambda genome. Inverted arrowheads and grey shading indicate regions of under- (regions 1 and 2) and over-representation (regions 3–5) of the lambda genome in the plume metagenome dataset. region 1, capsid; region 2, tail; region 3, integrase; region 4, superinfection exclusion; region 5, nin transcriptional terminators. (D) Mapping of surronding deep-sea metagenome reads to the lambda genome.

Their distribution and evenness of coverage, on the genome of the type-strain Enterobacteria phage lambda (GenBank accession NC_001416), was determined by mapping them to the lambda genome (Figure 3A). The lambda-like reads from the plume sample gave much higher coverage (average 5.04-fold, 14-fold coverage for 95.0%, 0 coverage for 12.1%, 5.7-fold coverage for all >0) of the lambda genome (Figure 3B) than those from the surrounding seawater sample (average 0.42-fold, 2 fold at 95th percentile, 0-coverage 72.7%, Figure 3C), although coverage in the plume sample was not even across the lambda genome (Figure 3, regions 1–5). The regions 3079–3999 nt and 11396–12956 nt on the lambda genome recruited no reads from either metagenomic dataset and correspond to the regions encoding the *B* capsid component (gi:2703526, Figure 3B, region 1) and the *H* tail fibre component (gi:2703511, Figure 3B, region 2), respectively. In contrast, regions of the lambda genome (35545–36309 nt and 40301–43323 nt) which encode the *rexB* protein and *nin* transcriptional terminators were highly covered by reads from the plume sample but not the surrounding seawater sample (Figure 3B, regions 4 and 5, respecively). We also observed good coverage of the region encoding the lambda integrase (27127–29899 nt, Figure 3B, region 3) by reads from both the plume and surrounding seawater datasets.

### Reconstruction of Lambda-like Genome by Shotgun-Assembly

We attempted to reconstruct a hypothetical lambda-like genome by assembling all reads from the plume sample using the Newbler software and by re-aligning the assembled contigs to the complete genome sequence of Enterobacteria phage lambda. The assembly resulted in 280 contigs (length >100 bp), of which 59 were large contigs with lengths greater than 800 bp (maximum length: 1859 bp, maximum recruited reads: 74).

Using exact global-local alignment (Needleman-Wunsch), 168 contigs including all large contigs align to the lambda genome with E-values<10^−3^. Fourteen out of all contigs exhibited a very high sequence identity (>99%) to a unique genomic region of the lambda genome, whereas all contigs with low to medium sequence identities (<75%) exihibited multiple other alignments of near equal E-value, score, and identity, indicating equivocal assignments to the reference genome (Best alignments locations are depicted in Suplementary Figure S2). Aligned contigs of high similarity enabled the reconstruction of several conserved lysogeny related genes such as *nin* and *int/xis* in the lambda genome (Figure S2). In general, the assembled contigs as well as unassembled reads showed an increased coverage towards the 3′ half of the genome. Based on assembled contigs 27.7% of the genome sequence is covered with >99% sequence identity, 53% of the genome is covered with >90% sequence identity, while 14150 bp (29%) are not covered by any contig at an E-value cut-off of 1e-3.

We tried to recover the sequence regions of zero coverage from the assembled contigs (*B* capsid component, *H* tail fibre components) using global-local alignments and exact local alignments (Smith-Waterman) of all contigs against the CDS. To calibrate our search we aligned the CDS sequences of tail protein *H* and capsid protein *B* against the lambda genome using glsearch, yielding multiple significant hits with extremely low E-values (minimum 7.2e-115, 1.4e-174 repectively) in other locations of the genome. Each gene region overlaps only with two weakly similar contigs (sequence identity 49.0%–64.7%) and no significant local alignment was discovered using Smith-Waterman search (ssearch36, E<0.001) indicating that low-coverage regions cannot be reconstructed from our sequences.

### Phylogenetic analysis

**Figure 4:**
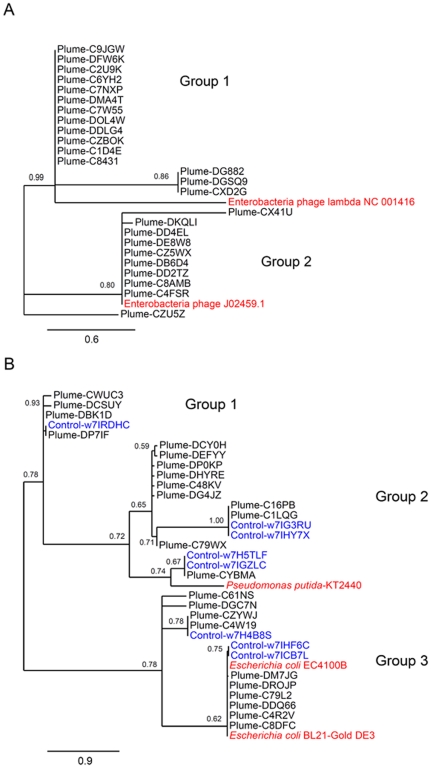
Phylogenetic analysis of lambda *rexB*-like and integrase-like metagenome sequences. Maximum likelihood analysis was used to analyse diversity of partial gene sequences present in metagenome reads using (A) *rexB* superinfection exclusion protein-like sequences present in the plume metagenome sample and (B) integrase-like sequences in the plume and surrounding seawater metagenome samples. Branches with support values lower than 50 are collapsed. Reference sequences, plume metagenome sequences and surrounding seawater metagenome sequences are shown in red, black and blue, respectively. Scale bar represents (A) 0.6 and (B) 0.9 substitutions per site.

The abundant recruitment of metagenome sequences to specific regions on the lambda genome scaffold could either be due to multiple copies of the same sequence, or it may reflect sequence diversity within specific genes in the lambda-like viral population. To distinguish between these possibilities, we performed phylogenetic analysis of the sequences which mapped to two regions of high coverage on the lambda genome: *rexB* Figure 4A) and lambda integrase (Figure 4B). For *rexB*-like sequences, we identified two distinct clusters (Groups 1 and 2) with a branch support of 0.80 and 0.99, respectively. Group 1 sequences were only distantly related to the *rexB* gene from one phage lambda sequence (GenBank accession NC_001416), while Group 2 showed a high sequence similarity with the *rexB* gene found in a second Enterobacteria phage lambda genome sequence (GenBank accession J02459). Lambda *int*-like sequences from both metagenomes fell into three distinct clusters, identified as Groups 1–3, all with branch supports greater than 70. Groups 2 and 3 show highest sequence similarity to phage integrases in *Pseudomonas putida* and *Escherichia coli* phages, respectively. There is no clear delineation between *int*-like sequences present in the plume and surrounding seawater metagenome datasets, although one clade (within Group 2) contained only sequences from the plume metagenome. Interestingly, two of the lambda integrase reference sequences share high sequence similarity with two sequences from the surrounding seawater sample, and with six sequences from the plume sample (Figure 4B).

## Discussion

We present here the results of a novel methodological approach to identify key viruses in a hydrothermal vent plume and surrounding seawater. Our approach has combined the resolving power of high-throughput metagenomic sequencing and bioinformatics techniques with targeted community sub-fraction analysis to reduce viral community complexity and aid in the extraction of biologically meaningful information about the habitat under investigation. The results from this study have enabled us to reconstruct a near-complete Enteriobacteriophage lambda-like virus “genome" from the greater virus community within a deep-sea hydrothermal plume and the deep ocean seawater surrounding it. Phylogenetic analysis of the gene sequence diversity present in this lambda-like micro-metagenome has provided an intriguing snapshot of the viral armoire and its potential role in fitness-driven adaptation in this unique microbiome.

### Method development

Preparation of samples for viral metagenomic investigation typically requires a combination of large sample volumes (100–200L) and amplification of viral nucleic acids [37]. Using a new method for constructing high quality viral DNA for metagenome sequencing, 21–30 Mb of DNA sequence were derived from filtration of only 40 litres of seawater. Prefiltration and concentration of viruses from seawater will always result in high losses of virus from the sample [40], [46]. As the purpose of the present study was not to fully map virus diversity in a hydrothermal plume, but to establish new methods for targeted identification of key viruses in the ecosystem, a slight reduction in viral diversity or virus abundance due to sample processing is not likely to alter the results or conclusions of the study. Nonetheless, unintentional reduction of the viral community by filtration was compensated for by two independent concentration steps (tangential-flow filtration followed by ultracentrifugation) followed by separation of the viral genomes from free DNA by PFGE. These secondary concentration/purification steps eliminated the need for additional sample-processing such as PEG-precipitation, density gradient ultracentrifugation, and enzymatic digestion of non-viral DNA, which normally reduce DNA yield and may introduce bias [37].

The appearance of a band on PFGE gels is direct evidence for its numerical dominance in the analysed virus concentrate (>1% of virus particles, [47], thus making this method ideal for targeted examination of key viruses present in complex viral communities. The non-disruptive preparation of agarose plugs for PFGE minimises unintentional fragmentation of viral genomes, therefore PFGE bands should accurately represent true viral genome sizes [40]. Despite sufficient concentration of individual size-classes of viral dsDNA genomes for visualization by PFGE, amplification of viral DNA was still necessary to generate sufficient template for metagenome sequencing. Several amplification methods have been developed for this purpose, each with unique amplification biases. Multiple displacement amplification (MDA) of environmental DNA has been reported to enrich for small, circular, ssDNA genomes [48], [49], also in marine samples [32], [50]. Another method for sample amplification generates a linker-adapted shotgun library (LASL) [34], in which specific oligonucleotide linkers are ligated to the ends of metagenome DNA fragments. PCR amplification from the linker sequences should result in non-biased amplification of all linker-adapted DNA fragments. This approach, which was used for the present study, was employed to circumvent amplification biases associated with the MDA protocols, as it exploits a more uniform priming, fewer rounds of PCR, and the pooling of separate reactions. Nevertheless, it is impossible to completely avoid amplification bias from PCR-based methods [51].

An alternative method for investigation of unknown viral communities is fosmid cloning, in which large environmental DNA fragments are captured and maintained in cloning vectors [52], [53]. This method possesses the advantage that genomic context is maintained in individual clones, thereby allowing examination of genomic content from single copies of viral genomes, which in the case of small viruses may cover the entire viral genome [54]. Similar to fosmid cloning, the present method facilitates potential reconstruction of entire viral “genomes" due to the deliberately-reduced sequence complexity in the sample. Fosmid cloning, however, is subject to cloning-related artifacts such as counterselection against potentially toxic genes often found in phage genomes, bias toward capture of specific-size inserts, influence of modified DNA often present in viral genomes, and the need for high concentration, molecular weight and quality of viral DNA [55], [56]. In addition, the generation and maintenance of a fosmid library with sufficient coverage of a complex viral community would be extremely challenging. The method presented here captures sequence diversity within a specific and targeted viral sub-community, and thus represents a satisfactory compromise between the particular advantages associated with either fosmid cloning or whole virome metagenomic sequencing.

Despite the decreased complexity inherent in our method, we did not observe significant deviation from previous studies with regard to relative proportions of unknown sequences in our viral metagenome samples, which were 77% and 79% for the plume and surrounding seawater samples, respectively, using BLASTX searches again the nr database (Table 1). The results of metagenomic studies of viral-fraction DNA from the marine environment [32], [34], [55], [57], [58] have repeatedly demonstrated that 65–99% of viral metagenome sequence data shows no similarity to database entries. Analysis of the viral fraction of marine sediments by Breibart and colleagues [57] revealed no significant (E-value< = 10−3) similarity for 75% of metagenome sequences when TBLASTX was used to search against GenBank), while another study by Breitbart et al. [34] found that 74% of sequences from a surface seawater viral metagenome had no significant hits to the databases. Similarly, Bench et al. [58] determined that 61% of sequences from a Chesapeake Bay virioplankton metagenome had no similarity to well-studied organisms. The resolving power of bioinformatics tools and the representativeness of sequences present in the databases, however, should improve with continued metagenome sequencing and virus-host system isolation efforts.

### Targeted examination of sub-community viral diversity

The observation that dsDNA virus with no RNA stage dominated the virus-like reads in both samples, in addition to shared dominance of these reads by Myovirus-like and Siphovirus-like sequences (Figure 1B), are consistent with the size-limited nucleic acid selection from total viral communities for metagenomic sequencing, and bespeak the similarity of viruses in the investigated habitats to previously described viral taxa. The size range of dsDNA in excised PFGE bands (31–48 kb) is within the size ranges reported for Caudovirales [59], making it unsurprising that Caudovirales were abundant among the virus-like sequences identified in our datasets. Analysis of the plume and surrounding seawater samples by mapping to the Phage Proteomic tree (Figure 2, Data S1) corroborated BLAST results (Figure 1) and also allowed informative visualisation of diversity and distribution of phage-like reads in our plume and surrounding seawater datasets (Figure 2). Although metagenomic reads from both datasets mapped to almost all taxa in the Phage Proteomic Tree, there were a few noteworthy Siphoviridae and Myoviridae taxa which recruited an abundance of reads, further underscoring the use of this method for reducing viral community complexity for identification of key or prominent viral taxa. It should be noted, however, that this observed dominance may be an artefact of database bias.

The fortuitous finding that lambda-like bacteriophage are unequally represented among Siphoviridae in the plume and seawater samples, both by BLAST analysis and by mapping to the Phage Proteomic Tree, combined with knowledge that the Siphoviridae family of viruses contains many temperate marine bacteriophage [43], and that there exists strong evidence for prevalence of lysogeny at hydrothermal vents [33] and in the deep ocean [45] led us to further examine the genomic content of this particular fraction of the viral community in an attempt to extrapolate information about the plume ecosystem through the genomic content of a key viral sub-community. Although similar cases of differential dominance between plume and surrounding seawater datasets were also visible for Myoviridae-like taxa (Figure 1B), these reads were not further investigated in the present study for reasons of conciseness. It should be emphasized that one of the clear advantages of method presented in this study is its utility for identification of potentially dominant viral taxa in samples delimited by both researcher-defined size ranges and taxonomic groups.

One of the challenges associated with conventional viral metagenome analysis is the daunting degree of sequence diversity and complexity in the resulting sequence library, which can greatly complicate the assembly process [32], [38]. Our targeted sequencing approach enabled us to forego the necessity for read assembly by limiting the amount of sequence information generated in a biologically meaningful way, i.e. by examination of only one size fraction of dsDNA viruses present in our samples. For these reasons, we were able to identify potentially important viruses from a diverse and poorly-understood viral community without the need for culturing or expansive computational capabilities. Assembly of plume reads for the specific purpose of mapping to the lambda genome did not reveal any particular benefit when compared to mapping with unassembled reads from the same dataset (Figure S2), nor did assembly permit closing of the un-mapped capsid and tail protein regions of the lambda-like phage “genome" in the plume sample (Figure 3B, regions 1 and 2).

### Gleaning virus lifestyle and function from micro-metagenome analysis: the case for lysogeny

The abundance of lambda-like virus sequences in both the plume and surrounding seawater metagenome datasets implies that these viruses may play a significant ecological role in the plume and surrounding seawater habitat. Enterobacteria phage lambda is a well-studied temperate bacteriophage of the gammaprotebacterium *Escherichia coli* [60]. The apparent abundance of an Enterobacteria phage-like viral community in the deep ocean and a hydrothermal plume is in accord with the findings of a concurrent study on prokaryotic diversity in the same plume and seawater samples, which revealed that the *Enterobacteriaceae* family of *Gammaproteobacteria* (51% of the bacterial clone library) dominated prokaryotic communities at the plume site (Reigstad et al, unpublished). In addition, genome sequence analysis of the thermophilic gammaproteobacterium *Thiomicrospira crunogena* XCL-2, isolated from a deep-sea hydrothermal vent, identified a lambdoid prophage in the host genome [61]. Lambda-like viruses have also been found in four marine metaviromes from distant geographic regions [32], [34].

Although the lambda-like “genome" described in the plume metagenome dataset was identified in the free-virus fraction of seawater, strong recruitment of viral micro-metagenome reads (Figure 3) led us to conclude that this particular viral “population" may represent a phage community fraction with both lytic and lysogenic potential. Viruses that are able to lysogenize or psuedo-lysogenize their hosts stand to gain the advantages of a) genomic stability during periods of host inactivity [62], [63], b) maintenance of a robust host population (in the case of pseudo-lysogeny; [64]), and/or c) protection from theoretically enhanced viral decay rates in chemically or thermally adverse environments [65], [66], [67], [68]. There is also evidence from studies of various host-bacteriophage systems that lysogenised bacteria may have elevated fitness compared to non-lysogens under specific growth conditions [69], or that lysogenic infection benefits host cells through immunity to superinfection [60]. It has been suggested that plumes and deep-sea conditions, such as those investigated in the present study, may select for viral lysogeny or pseudo-lysogeny due to fluctuation in bacterial production rates [33], [62], [70], [71], [72]. It is thus tempting to speculate that the lambda-like viral sub-community described here may represent the viral partner in a key and potentially mutualistic host-virus interaction that promotes survival in the challenging environment of the deep ocean and hydrothermal vent environment. Our study has therefore focused on the diversity of plume and seawater metagenomic reads with similarity to particular gene regions of the lambda genome in order to gain knowledge about the potential ecological and evolutionary processes driving adaptation in this key viral sub-community.

### Sub-genomic diversity within a lambda-like subcommunity and the host-virus “arms race"

Variation in competitive ability for limiting nutrients determines the composition of bacterial populations in the environment (bottom-up regulation, [14] and indirectly dictates the composition of the viral community. Although zooplankton grazing may be a source of prokaryote mortality in hydrothermal plumes [73], [74], [75] the purpose of this study was to identify key viruses involved in top-down predatory regulation of hydrothermal plume prokaryotic communities. Discussions of host-virus interactions frequently invoke speculation about the host-virus “arms race" [76], [77], [78], [79] by which host defenses and viral offensive strategies succeed each other in endless procession. What can the diversity of lambda-like bacteriophage genes tell us about the host-virus “arms race" in this hydrothermal vent plume system?

The notable lack of coverage by plume and seawater reads of the lambda genome regions encoding capsid (Figure 3B, region 1) and tail fibre (Figure 3B, region 2) proteins suggests that extensive sequence divergence at these loci has inhibited our ability to identify lambda-like sequences at these regions. The absence of identifiable tail fibre homologues in the lambda-like sub-community in the plume sample is consistent with viral attachment to hosts via uncharacterised and/or undescribed host receptors which bear no resemblance to lambda receptors in *E. coli*. Polymorphisms in tail fibre genes is a known viral strategy to overcome development of host resistance [80], thus it is plausible that the genes encoding these phage structural genes may have diverged to the point that they can no longer be recognised using bioinformatic predictions [61]. In addition, this serves to further emphasize that, although the “bacteriophage genome" assembled here shares remarkable sequence similarity with Enterobacteria phage lambda, it is clearly both polymorphic and distinct from lambda.

The absence of lambda-like capsid gene sequences is somewhat more difficult to interpret in the context of an “arms race", as viral capsids are not known to mediate host-virus interactions. Variability in capsid genes might have arisen from diversifying selection in order to optimize the tolerance of the physical capsid structure to the physicochemical conditions in hydrothermal vent systems or in the deep ocean. It is also quite possible that host-virus interactions in these environments occur by novel recognition and binding pathways or mechanisms that bear little or no resemblance to described mechanisms. The limited information available for hydrothermal vent systems in general cannot eliminate this scenario. The lack of lambda capsid-like sequences in our metagenomic samples also raises questions about the capturing power of primers for major capsid protein genes in studies of viral community diversity in environmental samples [62], [81]. According to our results, partial or entire populations of virus may be missed using such methods.

The RexAB system of lambda inhibits superinfection [82], [83], by altering the physiology of the host cell to prevent replication of incoming virus infections [84], [85]. RexB is thought to form an ion channel in the host cell membrane that, upon activation by RexA during superinfection, destroys the cellular membrane potential, thereby eliminating the ability of the cell to function as a viral factory for the superinfecting virus [85]. The real diversity of plume sample *rexB*-like sequences, as confirmed by phylogenetic analysis (Figure 4A), may manifest in variations in the ability of the RexB-like gene product to inhibit multiple viral infections of the same host cell, with the potential consequence of altering the success of virus infection and, thereby, the structure of the viral community at this site.

Integrases, or integration proteins, are viral enzymes that mediate integration of the virus genome into the host genome through site-specific unidirectional recombination between the viral *attP* and host *attB* attachment sites [86]. Certain polymorphisms in integrase enzymes may increase fitness of temperate phage by fine-tuning the specificity of *att* site recognition and the efficiency of integration [86]. The integrase gene has been shown to be a useful phylogenetic marker for the environmental analysis of temperate phages [87]. It has also been shown to give good phylogenetic resolution when resolving relationships between closely related taxa [88]. In our study, a phylogenetic investigation of the integrase sequences present in the plume sample (Figure 4B) revealed notable gene diversity but with three clear groups which is consistent with our hypothesis that sequence variation at this locus may indeed reflect selected modulation of host-virus interactions.

The region of the lambda genome with highest coverage by the plume sample reads coincides with the lambda *nin* region transcriptional terminators (Figure 3B). Lambda maintains tight control over polycistronic gene expression, and thereby lytic and lysogenic gene expression programmes, through the use of transcriptional terminators and anti-terminator proteins. The anti-terminator lambda N-protein overrides transcriptional termination signals present in the *nin* region, allowing expression of lambda genes required for entry into the lytic cycle [89]. Sequence divergence at the *nin* region may therefore reflect attempts to modulate the strength or efficiency of N-binding for greater control over viral “decisions" to initiate late gene expression and prophage induction (i.e. entry into the lytic cycle), e.g. in response to rapid fluctutations in nutrient availability.

Optimisation of superinfection exclusion, virus integration and lysis/lysogeny “switch" functions provides clear fitness advantages for lyosgenic phages. The ability of bioinformatics tools to identify superinfection exclusion, integration, and transcriptional regulatory elements suggests that, despite some sequence variability, these genes/regions are evolutionarily constrained, making them thus accessible to identification given the current knowledge base for isolated viruses and their genome content.

The diversity of virus genes in our lambdoid virus “population", as a genetic record of the host-virus arms race in hydrothermal vent plumes, might be mirrored by complementary diversity in the co-existing host population. Isolation and characterization and/or metagenomic analysis of prokaryote populations in hydrothermal vent systems would further aid in identification of key viruses in these environments by providing information about prophages and host metabolic pathways. As stated above, viruses mediate considerable genetic exchange via transduction, and functional genes of clear host origin are not uncommon in virus genomes and populations [81], [90], [91]. Viromes are therefore good hunting grounds for host genes that confer adaptive advantages for viruses [7]. In addition, further efforts to isolate and characterise host-lambdoid virus systems from samples such as those examined in this study might contribute greatly to our understanding of lysis/lysogeny switches and the magnitude of their impact on host-virus dynamics in hydrothermal vent plumes.

### Influence of surrounding water mass on plume composition

Discovery of the Loki's Castle vent field (2400 m depth) in 2008 provided a unique opportunity for investigation of microbial community composition and dynamics, including host-virus interactions, in an Arctic hydrothermal vent plume [39]. A key question regarding the biochemical processes in deep-sea hydrothermal plumes is whether the microbial communities in the plumes are indigenous or if they are opportunistic immigrants from the surrounding seawater. Recent evidence has shown that the microbial diversity and community structure were remarkably similar in the background and plume water [19]. In our study, the viral communities both in the plume and the near-vent environments include lambda-like bacteriophage, although represententation of this lambdoid phage population was much stronger in the plume sample. Hydrothermal plumes consist primarily (∼99%) of deep ocean seawater mixed with hydrothermal solution (∼1%) in a ratio that varies greatly with distance from the hydrothermal vent source [19]. One problem that remains unsolved is whether the hydrothermal solution enriches for specific groups of microorganisms. Our findings corroborate the theory that the biological component of plume water is indeed influenced by the surrounding deep ocean seawater. Given the targeted focus of the present study, it is possible that viral groups endemic to the hydrothermal plume were simply not detected using the present methodology.

### Conclusions

Despite an extremely high diversity in the studied environment, our sampling and analysis approaches have enabled us to extract biologically significant genetic information describing key members of viral communities in the deep ocean. Metagenomic examination of prominent, genome size-delimited viral community fractions from the hydrothermal plume sample allowed the near-complete assembly of a lambdoid phage genome, and in addition identified an intriguing sub-genomic diversity within the lambda-like phage “population". While our methodology has allowed us to focus on one apparently prominent viral population, any comprehensive consideration of viral regulation of microbial community composition and function would need to take into consideration the entire diversity of viruses and hosts present in hydrothermal vent plumes. Unveiling the diversity of the viral gene pool through both culture-dependent and culture-independent research will thus be vital for advancement of our understanding of viral-host interactions in marine environments. Although the neither the biology nor the ecology of the viral population can be conclusively determined from these analyses, the uniqueness of the plume ecosystem in combination with strong evidence for viral lysogeny provides tantalizing material for future exploration of host-virus interactions in under-explored deep sea environments.

## Materials and Methods

### Sample collection and processing

Waters from the deep-sea hydrothermal plume above the arctic high-temperature Loki's Castle vent field [39] and the surrounding deep seawater sample were collected during the R/V G.O. Sars cruise to the Knipovich Ridge in 2009. The plume was identified using a Seabird conductivity/temperature/depth (CTD) profiler equipped with a particle sensor (C Star Transmissometer), and an Eh-sensor. The hydrothermal plume water (73°33.97′N; 08°09.51′E; “plume") was collected at a depth of 2201 m, and a sample from the seawater 50 km distal to the plume (73°10,37′N; 08°56.52′E; “seawater") at a depth of 2100 m. The samples were collected using the CTD rosette fitted with 24×10L Niskin bottles.

Forty litres of seawater per sample was pre-filtered through a 142 mm diameter 1.2 µm pore-size low-protein-binding Durapore membrane filter (Millipore Corp., Billerica, MA, USA) then filtered through a 0.22 µm Sterivex filter (Millipore Corp., Billerica, MA, USA) to remove zooplankton, phytoplankton and bacteria from the sample. The virus-containing filtrates were further concentrated to a final volume of approx 100 ml by tangential flow filtration using a QuixStand benchtop system equipped with a 100.000 pore size (NMWC) hollow fibre cartridge (GE Healthcare Bio-Sciences AB, Uppsala, Sweden). Recovery of the viruses using this approach has been measured to be between 40–60% (Sandaa, personal observation, [40], [46]. Virus concentrates were stored at 4°C in the dark until further processing.

### Viral and bacterial counts

Total number of bacteria and viruses in unfiltered plume and surronding deep-sea seawater samples were determined with a FACSCalibur flow cytometer (Becton–Dickinson) equipped with an air-cooled laser providing 15 mW at 488 nm and with standard filter set-up. Enumeration of virus-like particles and bacteria was performed on samples fixed with glutaraldehyde (final concentration 0.5% v/v) prior to staining with 1 X SYBR Green I. A minimum of two different dilutions per sample were counted for 60 s each at a viral event rate between 100 and 1000 s−1. The flow cytometer instrumentation and general methodology followed the recommendations of Marie [92], [93].

### Pulsed field gel electrophoresis

PFGE was performed according to the methods of Sandaa [40]. Briefly, viruses in 34 ml of virus concentrate were further concentrated by ultracentrifugation (Beckman L8-M with SW-28 rotor) for 2 h at 28,000 rpm at 10°C. The viral pellet was dissolved in 200 µL of SM buffer (0.1 M NaCl, 8 mM MgSO4·7H2O, 50 mM Tris-HCl, 0.005% (w/v) glycerin). Two viral agarose plugs were prepared from the 200 µL viral concentrate for PFGE. Lysis of the viral particles was performed in freshly made lysis buffer (250 mM EDTA pH 8.0, 1% SDS, 1 mg/ml Proteinase K). The agarose plugs were run on a 1% w/v SeaKem GTG agarose (FMC, Rockland, Maine) gel in 1X TBE gel buffer using a Bio-Rad DR-II CHEF Cell (Bio-Rad, Richmond Ca, USA) electrophoresis unit. The two plugs were run at two different pulse-ramp conditions in order to separate a large range of viral genome sizes [94]. Gels were visualized and digitized using the Fujifilm imaging system, LAS-3000, and bands of interest were excised and frozen at −80°C.

### DNA extraction, amplification and pyrosequencing

DNA was eluted from the PFGE agarose gel slices in 10,000 MWCO Spectra/Por, Regenerated Cellulose dialysis membranes (Spectrum Laboratories Inc.CA, USA) by electrophoresis in 1 X TAE buffer (40 mM Tris-HCl, 1 mM EDTA, 40 mM acetic acid, pH 8.0) for 3 h at 70 V. Further concentration of the DNA was performed using Vivaspin 500 columns (Milipore Corp) according to the manufacturer's protocol.

The dominant PFGE band in each of the surronding deep-sea and plume samples (Figure S1) was selected for sequencing. Eluted DNA from these bands was amplified based on a linker-adaptor PCR method using the WGA1 and Genome Plex WGA reamplification kit from Sigma (Sigma Aldrich, St Louis, MO, USA). Six separate WGA reactions were run for each sample and pooled before further processing. The amplified products were purified using the GenElute PCR Clean-Up Kit (Sigma Aldrich) and stored at −80°C until sequencing.

Pyrosequencing was performed by LCG Genomic GmbH (Berlin, Germany) using the Roche/454 GS FLX Titanum pyrophosphate sequencing platform (Basel, Switzerland). The total amount of sequence data obtained was 21 Mb from the plume sample and 30 Mb from the seawater sample.

### Blast Database Search

Sequencing reads were subjected to a database search against the NCBI non-redundant sequence database (nr), environmental sample proteins from WGS projects database (env_nr), and one boutique nucleotide database containing all viral RefSeq Genomes (viral_refseq) annotated with NCBI taxon-ids using the Dust algorithm for low-complexity filtering. Databases were downloaded from the NCBI FTP site (ftp://ftp.ncbi.nih.gov/blast/db/) on December 29, 2011. BLASTX searches against the nr and env_nr and TBLASTX searches against viral_refseq were performed for metagenomic reads [95]. Blast results with E-values
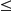
10^−3^ in at least one HSP were retained for further analysis. BLAST databases and result files were processed using Perl scripts and BioPerl modules [96].

### Taxonomic Analysis

Taxonomic composition was analysed using MEGAN4 [97] using the least-common-ancestor algorithm with a minimum BLAST-score of 35 to map sequencing reads to the NCBI-taxonomy, using nr results on the super-kindom level, and viral_refseq results below this level. For comparation of taxonomic profiles in MEGAN read counts were normalized to 100,000 reads per sample.

### Phage Proteomic Tree

Counts of the best blast results from TBLASTX queries against the refseq_viral boutique database were projected onto the Phage Proteomic Tree (PPT) v.6 as described in [42]. PPT data was obtained from http://phantome.org/PhageProteomicTree/latest/ in Nexus format and manually curated replacing generic taxon-identifiers with NCBI-taxonids (Data S2). The curated tree data are available in the supplementary material. Tree visualization was performed using the Interactive Tree of Life (ITOL) web-application [98]

### Sequence Assembly

*De-novo* assembly of 454-reads from the Plume sample was performed using the Roche/454 GS de novo assembler 2.5 (Basel, Switzerland) with minimal overlap identity of 95% and minimum overlap length of 20 bp.

### Recruitment of Reads and Contigs to Lambda Genome

The long-read component (BWA-SW) of the Burrows- Wheeler Alignment Tool [99] was used with default parameters to align all the sequencing reads to the whole genome sequence of Enterobacteria Phage lambda. The output of BWA-SW was converted to binary alignment format (BAM) using SAMtools [100]. Coverage plots were generated from the BAM files using R, Bioconductor [101] and the package GenomeGraphs [102]. Exact global-local nucleotide alignments of all assembled reads against the Enterobacteria phage lambda genome (RefSeq accession NC_001416.1) were computed using the Needleman-Wunsch algorithm [103] in glsearch36 from the FASTA alignment tools version 3.6 [104]. Exact local alignments of all reads against the full coding sequences, plus 100 bases wide flanks, of the two genes *B* (capsid component, gi:2703536) and *H* (tail component, gi2703511) were computed using the Smith-Waterman [105] algorithm in ssearch36. Exact global-local and local alignments were firstly retained up to an E-value of 10, secondly alignments were filtered and inspected at various levels of significance and score.

### Phylogenetic Analysis

The phylogenetic tree of partial *rexB*-like genes in the plume metagenome sample was constructed from 25 nucleotide sequences (Figure 4A) using two lambda *rexB* sequences (GenBank accession numbers NP_001416 and J02459) as reference. The integrase tree was calculated based on 24 partial *int*/*xis*-like nucleotide sequences from the plume sample and eight nucleotide sequences from the seawater sample (Figure 4B). Reference *int* gene sequences from three viruses (Genbank accession numbers EFW75790, ACT29922, NP_743689) were included in the analysis. Phylogenetic relationships were inferred from multiple alignments for the integrase (size range 158–492 nt) and *rexB*-like genes (size range 152–469 nt) using the CLUSTALX alignment tool [106] hosted at http://phylogeny.fr [107]. Phylogeny was analysed using the PhyML 3.0 program [108]. Supports for clades were estimated by the approximate likelihood-ratio test with SH-like settings. Branches with lower support than 50 were collapsed. Trees were drawn using the TreeDyn program [109].

## Supporting Information

Figure S1 **Pulsed-field gel electrophoretic (PFGE) assessment of genome sizes of key double-stranded DNA (dsDNA) viral fractions present in the plume and surrounding seawater samples.** The PFGE gel image shows key viral genomes present in the concentrated viral fraction sampled from Loki's Castle hydrothermal plume (L-1 and L-2) and from surrounding seawater (C). PFGE bands that were excised and processed for metagenome sequencing are indicated by boxes. M1 and M2, dsDNA molecular weight markers (in kilobases).
(TIF)

Figure S2 **Alignment of Enterobacteria phage lambda-like metagenome sequence reads to the lambda genome.** Best alignments (E-value≤0.001) of assembled contigs from the plume sample against the lambda genome using exact global-local alignments from glsearch36. Contig alignement positions appear grouped by increasing sequence similarity. From bottom to top: very low (white, <50%), low (grey, 50–75%), high (yellow, 75–99%), very high (red, >99%), coverage of unassembled reads (orange), selected CDS.
(TIF)

Data S1 **Full SVG image file of the Phage Proteomic Tree as produced by ITOL.**
(SVG)

Data S2 **Reconstructed and manually curated Phage Proteomic Tree v6 in NEXUS format with NCBI-taxonids as it was used in our analysis.** The tree was generated from the tree data at http://phantome.org/PhageProteomicTree/latest/ and annotation data from http://www.phantome.org/phage_metadata.html.
(TREE)
