# Supplementary figures and images for: Finding a Needle in the Virus Metagenome Haystack - Micro-Metagenome Analysis Captures a Snapshot of the Diversity of a Bacteriophage Armoire
Source: PLoS One. 2012 Apr 11;7(4):e34238. doi: 10.1371/journal.pone.0034238 (PMC3324506; doi:10.1371/journal.pone.0034238)

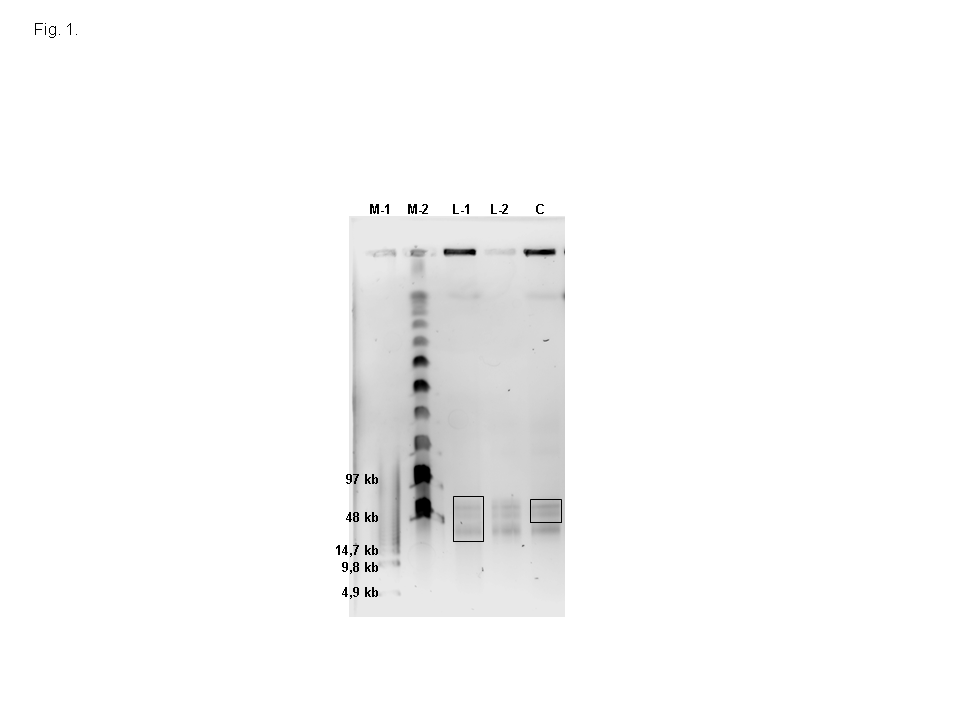

Supplement: Figure S1 — Pulsed-field gel electrophoretic (PFGE) assessment of genome sizes of key double-stranded DNA (dsDNA) viral fractions present in the plume and surrounding seawater samples. The PFGE gel image shows key viral genomes present in the concentrated viral fraction sampled from Loki's Castle hydrothermal plume (L-1 and L-2) and from surrounding seawater (C). PFGE bands that were excised and processed for metagenome sequencing are indicated by boxes. M1 and M2, dsDNA molecular weight markers (in kilobases). (TIF) [file pone.0034238.s001.tif]

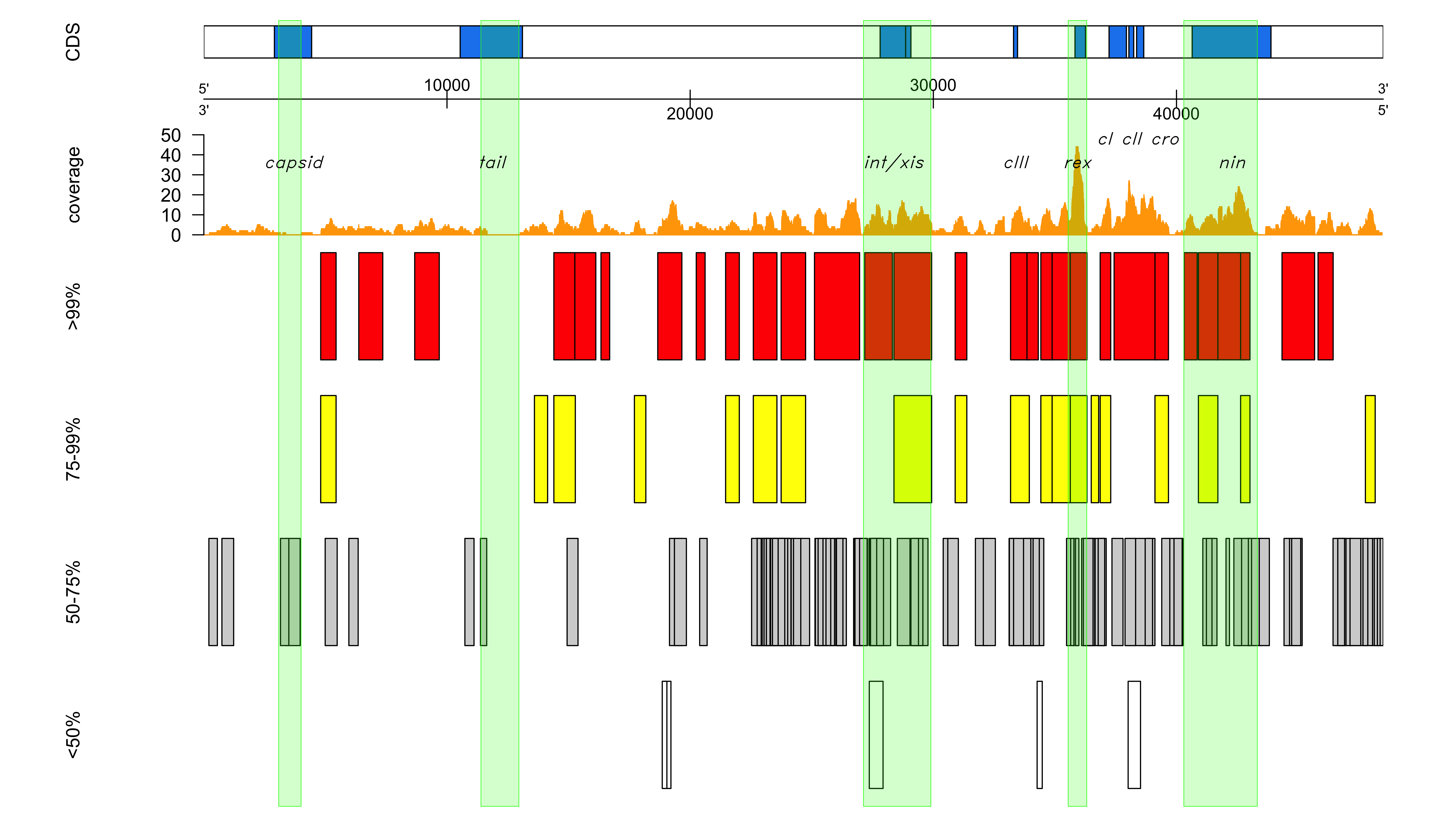

Supplement: Figure S2 — Alignment of Enterobacteria phage lambda-like metagenome sequence reads to the lambda genome. Best alignments (E-value≤0.001) of assembled contigs from the plume sample against the lambda genome using exact global-local alignments from glsearch36. Contig alignement positions appear grouped by increasing sequence similarity. From bottom to top: very low (white, <50%), low (grey, 50–75%), high (yellow, 75–99%), very high (red, >99%), coverage of unassembled reads (orange), selected CDS. (TIF) [file pone.0034238.s002.tif]
